# Epidemiology of *Helicobacter pylori* in Australia: a scoping review

**DOI:** 10.7717/peerj.13430

**Published:** 2022-05-31

**Authors:** Jillian Congedi, Craig Williams, Katherine L. Baldock

**Affiliations:** 1 UniSA Allied Health and Human Performance, Australian Centre for Precision Health, University of South Australia, Adelaide, South Australia, Australia; 2 UniSA Clinical and Health Sciences, Australian Centre for Precision Health, University of South Australia, Adelaide, South Australia, Australia

**Keywords:** Helicobacter pylori, Epidemiology, Prevalence, Australia, Scoping review

## Abstract

**Background:**

*Helicobacter pylori* (*H. pylori*), a bacterium implicated in the development of peptic ulcer and gastric cancer, is estimated to infect around half the world’s population. Its prevalence in Australia is unclear. This scoping review aimed to evaluate all Australian literature providing estimates of the prevalence of *H. pylori*.

**Methods:**

Australian studies examining *H. pylori* prevalence from 1982 onwards were eligible for inclusion. Medline, Embase and Scopus databases, and grey literature sources, were searched. Two independent reviewers undertook a two-stage screening process. Data were extracted by two independent reviewers using a pre-specified template.

**Results:**

Of 444 identified studies, 75 were included in the review. *H. pylori* prevalence in Australian population-based studies (*n* = 8) ranged from 38.0% in 1991 to 15.1% in 2002; however, estimated prevalence across all non-clinical population studies in diverse sub-groups (*n* = 29) has varied dramatically. Decreased prevalence has been more marked in populations with gastrointestinal symptoms and conditions compared to non-clinical populations. Data on *H. pylori*prevalence in vulnerable populations are lacking.

**Conclusions:**

This is the first scoping review of Australian studies reporting *H. pylori* prevalence. A wide range of study designs, population groups, geographic regions, and diagnostic methods was included, involving data collected over a 50-year period (1969 to 2018). The summary of *H. pylori* prevalence estimates over time in this review points to a decrease in prevalence in Australia, particularly among populations with gastrointestinal symptoms and illnesses; however, it is unknown whether there is inequity in prevalence trends across vulnerable sub-groups of the Australian population. Future research and interventions supporting the health and wellbeing of vulnerable populations is required to ensure equitable health gains are made for all.

## Background

*Helicobacter pylori* (*H. pylori*), a spiral-shaped bacillus, is a major risk factor for the development of peptic ulcers, some forms of gastric cancer and mucosa-associated lymphoid tissue (MALT) lymphoma (Kusters, van Vliet & Kuipers, 2006). There is also some evidence of an association with conditions such as cardiovascular disease and ischaemic stroke, although this is debated (Jiang et al., 2017). When used alongside standard treatment, *H. pylori* eradication therapy, typically comprising combinations of two to three antibiotics and a proton-pump inhibitor (Chey et al., 2017), can support healing of peptic ulcersand prevent their recurrence (Ford et al., 2016), and can reduce the risk of gastric cancer (Shiotani, Cen & Graham, 2013; Chiang et al., 2021).

*H. pylori* is an important pathogen from a public health perspective. It is estimated that in 2018, around 800,000 new cases of gastric cancer worldwide could be attributed to *H. pylori* infection (de Martel et al., 2020). The 2010 Global Burden of Disease Study estimated that 3.5 deaths per 100,000 population per year were due to peptic ulcer disease (Stewart et al., 2014), for which *H. pylori* is a major risk factor (Kusters, van Vliet & Kuipers, 2006; Kuipers, Thijs & Festen, 1995). In addition, studies of Japanese-American men have found *H. pylori* infection to be associated with 3.0 to 4.7 times the odds of developing peptic ulcer disease compared to those uninfected (Nomura et al., 2002; Nomura et al., 1994).

There is evidence to suggest that *H. pylori* prevalence varies according to place, person and time characteristics. A systematic review of global *H. pylori* prevalence estimated that, in 2015, there were approximately 4.4 billion individuals with *H. pylori* infection worldwide (Hooi et al., 2017). This review of prevalence data from 62 countries reported large differences in *H. pylori* prevalence across geographic regions, with the highest prevalence in Africa (70.1%; 95% CI [62.6–77.7]) and the lowest prevalence in Oceania (24.4%; 95% CI [18.5–30.4]) (Hooi et al., 2017). Another systematic review of global *H. pylori* prevalence reported wide variation in prevalence between countries, from 13.1% in Finland to 90% in Mexico (Peleteiro et al., 2014). Studies have similarly shown variation across sub-populations within countries, typically with higher *H. pylori* prevalence in vulnerable groups such as migrant and Indigenous populations (Jones et al., 2012; Windsor et al., 2005). For instance, the systematic review by Hooi et al. (2017) reported that, in Australia, the general population pooled prevalence was 24.6% (95% CI [17.2–32.1]) compared to 76.0% in a rural Western Australian Indigenous community. The same systematic review reported a pooled prevalence estimate in the United States of 35.6% (95% CI [30.0–41.1]) for the general population, compared to 74.8% (95% CI [72.9–76.7]) in an Alaskan Indigenous population. In addition, there are documented associations between poorer social and environmental contextual factors, for example low socio-economic status and crowded living conditions, and *H. pylori* infection (Mentis, Lehours & Mégraud, 2015; Cheng et al., 2009; Genta, Turner & Sonnenberg, 2017; Lim et al., 2013; Pandeya, Whiteman & Australian Canc Study, 2011), and numerous studies have found that *H. pylori* prevalence increases with age (EUROGAST Study Group, 1993; Lane et al., 2002; Megraud et al., 1989).

With regard to variations in prevalence over time, systematic reviews have reported that the population-wide prevalence of *H. pylori* may have decreased in some, typically more industrialised, countries in recent decades. Hooi and colleagues reported lower period prevalence from 2000–2016 compared to 1970–1999 in Europe, North America and Oceania, whereas similar prevalence across the two time periods was reported for Asia, Latin American and the Caribbean (Hooi et al., 2017). In contrast, Nagy, Johansson & Molloy-Bl (2016) reported a decrease in *H. pylori* prevalence in China from 1983 to 2013 (25 studies; 28 datasets), but no significant trend was observed in prevalence over time for the United States (1990–2006; 11 studies). It has been stated that higher prevalence of *H. pylori* with increasing age is likely due to a cohort effect rather than incremental infection over the life course (Banatvala et al., 1993). Mitchell & Katelaris (2016) have argued that this cohort effect, whereby each birth cohort has a lower overall *H. pylori* prevalence than the cohorts before them, has led to a decrease in the prevalence of infection in Australia over time.

In Australia, estimated population prevalence has ranged from 38% in 1991 (Lin et al., 1998a) to 15% in 2002 and 2005 (Pandeya, Whiteman & Australian Canc Study, 2011; Moujaber et al., 2008). However, these prevalence data represent different population groups and different age ranges. The earlier study from data collected in 1991 (Lin et al., 1998a) included 273 participants aged 20–80 years from the Melbourne metropolitan area; a sample which is unlikely to be representative of the Australian population as a whole. The two later studies included larger samples from across Australia. One utilised a random sample of 2,413 serum samples from 37 major diagnostic laboratories across Australia, collected from people aged from 1 to 59 years (Moujaber et al., 2008). The other study used data from 1,355 community controls aged 18 to 79 years, who were recruited for a nation-wide case-control study of oesophageal cancer (Pandeya, Whiteman & Australian Canc Study, 2011).

There have been no studies published to date which have comprehensively reviewed and reported on studies of the prevalence of *H. pylori* in Australia over time. Given the public health importance of *H. pylori* infection, even in populations with relatively lower prevalence such as Australia, and lack of existing reviews of Australian *H. pylori* prevalence, this scoping review aimed to systematically identify and describe all studies reporting prevalence of *H. pylori* in Australia. In particular, this scoping review aimed to describe the scope of Australian *H. pylori* prevalence studies in terms of study characteristics (*e.g.*, geographic location, population and diagnostic methods), and to describe prevalence estimates according to person (*e.g.*, type of population studied, diagnoses, age, gender) and time characteristics (year(s) of data collection relating to *H. pylori* status).

## Methods

This review was performed in accordance with the guidelines set out in the PRISMA Extension for Scoping Reviews (Tricco et al., 2018).

### Eligibility criteria

All studies reporting prevalence of *H. pylori* infection in Australian populations from 1982 onwards were eligible for inclusion. The search was limited to studies published from 1982, when *H. pylori* was first identified. Reviews, letters, commentaries or opinion papers were excluded. Studies were also excluded if they reported on a dataset that was published in a more recent or complete study.

### Information sources

Medline, Embase and Scopus were searched for articles published from 1982 onwards on 26/06/2017 (search updated on 29/01/2021 to capture additional studies published between the original and updated search). Reference lists of the included studies were hand-searched to identify any additional relevant studies. Grey literature was searched using Google, Web of Science for conference presentations, and online government sources including the Australian Bureau of Statistics (ABS), the Australian Institute of Health and Welfare (AIHW) and the State Health Departments. A search was made on websites of all Australian universities to find researchers who conduct *H. pylori* research*.* These researchers (some of whom were authors of included papers), were contacted by email for information about current research, unpublished studies or studies not identified by previous searches.

### Search strategy

The search strategy was developed in conjunction with an experienced University of South Australia librarian. The search was performed using the following search terms together with relevant Boolean Operators and MeSH terms identified for individual databases: *Helicobacter pylori* (*Helicobacter pylori* *or *H*? *pylori* * or *Campylobacter pylori* *), Australia (australia* or tasmania* or victoria* or new south wales or queensland* or northern territory* or christmas island or canton island or enderbury island or melbourn* or sydney or adelaid* or perth or hobart or canberra or brisbane or darwin), Prevalence (prevalen* or infection rate* or proportion* or frequenc* or occurrence* or likelihood* or probabilit*), Epidemiolog*, risk factors (“population? at risk” or risk factor?), cohort studies (follow up stud* or follow?up stud* or longitudinal stud* or longitudinal survey* or prospective stud* or retrospective stud* or cohort stud* or cohort analys?s or con?current stud* or incidence stud* or cross?section* stud*), population surveillance (Population Surveillance or Sentinel Surveillance or Public Health Surveillance or general population* or screen*), asymptomatic infections (a?symptomatic infection* or sub?clinical infection*). See Appendix 1 for full search details.

### Study selection

Search results were imported into Endnote (The EndNote Team, 2013) where duplicates were removed. The studies were then imported into Covidence (Covidence, 2022) and were screened in duplicate by two independent reviewers (JC and KB) through a two-stage process: (1) screening titles and abstracts; and (2) reviewing full text of articles identified in step 1). Any differences were discussed between the reviewers (JC and KB) to reach consensus.

### Charting the data

The data extraction process was completed by two independent reviewers (JC and KB) using a standardised template. Any differences were resolved through discussion among the review team. The following information was extracted from the selected papers: “title”, “authors”, “year of publication”, “location of study”, “study design”, “year(s) of data collection”, “*H. pylori* testing method(s) used”, “description of study population”, “age groups”, “sample size”, and “*H. pylori* prevalence (percentage)”.

### Collating and summarising the results

Data were categorised according to study design and then tabulated in chronological order according to the date(s) of data collection. Studies for which no data collection date was available were listed chronologically by date of publication. The data were described in terms of types of populations studied, diagnostic methods used, Australian state and *H. pylori* prevalence. Study results were also analysed using meta-regression to estimate trends in *H*. *pylori* prevalence over time in clinical populations (those with gastrointestinal symptoms or conditions) and non-clinical populations. Prevalence data organised by clinical and non-clinical populations were plotted using the method described by Nagy, Johansson & Molloy-Bl (2016).

## Results

**Figure 1:**
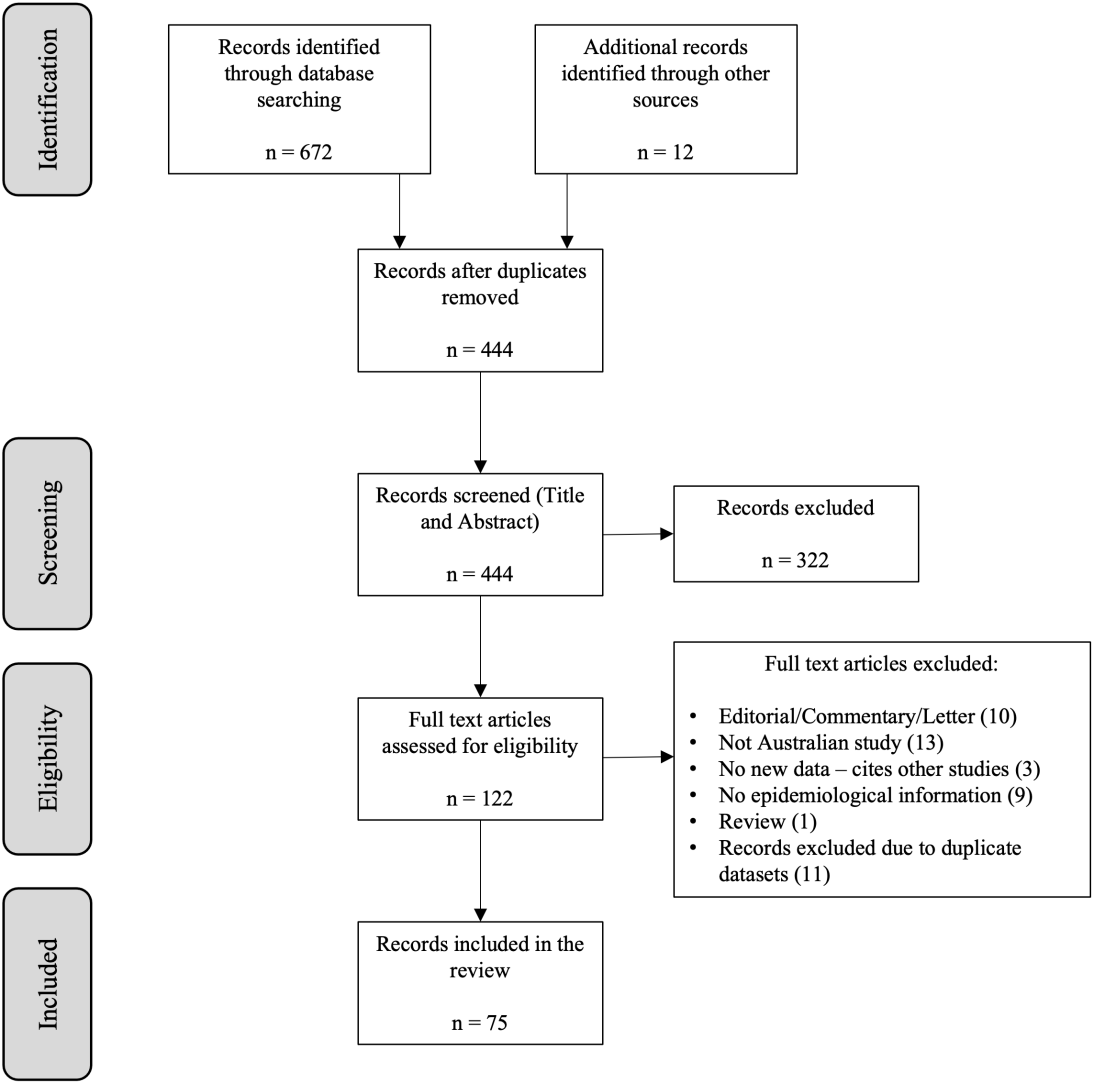
Flowchart illustrating the process of selection of papers for inclusion in the review.

The search resulted in 86 publications that met the inclusion criteria. Of these, 75 distinct studies were included in the review (Fig. 1).

The included studies were published between 1988 (Dwyer et al., 1988a; Dwyer et al., 1988b; Mitchell et al., 1988) and 2020 (Chamberlain et al., 2020; Endall et al., 2020) and were based on data collected between 1969 (Cullen et al., 1993) and 2018 (Sharma & Dowling, 2018; van der Poorten et al., 2018). Although *H. pylori* was identified in 1982, some studies used frozen plasma from earlier studies to determine *H. pylori* prevalence prior to 1982. Eleven of the 75 papers were conference abstracts for which no subsequent published paper was found. The majority of studies used a cross-sectional design. Characteristics of included studies are presented in Table 1.

**Table 1:** Study characteristics.

Study author & publication date; State; Year(s) of data collection	Diagnostic method	Study population or condition	Age	*n*	*H. pylori* Prevalence (%)
**STUDY DESIGN: PROSPECTIVE COHORT**					
Coles et al., 2003 Western Australia 1981	Serology	- All participants - CHD - Stroke	40–89 Mean 59.0 Mean 65.5 Mean 67.8	451 218 119	57.6 64.2 60.5
Dugué et al., 2019 Victoria 1990-1994	Serology (Immunoblot)	- Gastric cancer - Controls	Median: 62	159 159	77.0 60.0
Borody et al., 1994 New South Wales not stated	Urea Breath Test	Previously diagnosed *H. pylori* infection	24–82	94	2.2
Eslick et al., 2002 New South Wales not stated	Serology	Pregnant women	15–44	448	19.9
Chung & Cummins, 2009 (Conference abstract) South Australia not stated	Serology Testing of biopsy tissue (method not stated)	Gastritis	18–85	78	19.2
Pringle et al., 2015 New South Wales not stated	Serology	- Pregnant Aboriginal women - Blood donors (controls)	13–40	131 150	33 3
**STUDY DESIGN: RETROSPECTIVE COHORT**					
Cullen et al., 1993 Western Australia 1969, 1978 & 1990	Serology	Random selection from Busselton Health Survey.	1969: 20.2–44.0 1978: not stated 1990: 41.2–64.0	141 110 141	39.0 40.9 34.8
Mitchell et al., 1993a New South Wales 1971 & 1987–1991	Serology	1971: - Hepatitis positive children - Family members of *Hp+* children - Family members of *Hp-* children 1987–1991: - Family members of 21 *Hp+* children - Controls for family members	3–17 7–59 10–53 1–49 1–49	17 21 40 69 69	35.2 76.1 15.0 76.8 21.7
Lambert et al., 1995 Victoria 1977 & 1989	Serology	Institutionalised adults: - 1977 - 1989 - Community controls- 1989	not stated	122 122 122	34.4 75.4 23.0
Schimke et al., 2009 Western Australia 1993–1996	Serology	Diabetes	62.0 ± 13.3	1301	60.6
Mangira et al., 2014 (Conference abstract) South Australia 2012–2013	Rapid Urease	Endoscopy patients	58 ± 1	400	14.5
**STUDY DESIGN: CASE-CONTROL**					
Hardikar et al., 1996 Victoria 1990–1991	Serology	- Children with RAP - Controls	5–12	98 98	5.1 14.3
Chamberlain et al., 2020 Victoria 1990–1994	Serology	Gastric cancer cases Controls	Median 61 (IQR 56–65) Median 62 (IQR 56–65)	168 163	84.0 64.0
Whiteman et al., 2010 National 2002–2005	Serology	- Esophageal adenocarcinoma - Esophagogastric junction adenocarcinoma - Esophageal squamous cell carcinoma - Community controls	18–79	269 307 218 1355	13.0 12.1 24.8 22.3
Thrift et al., 2012 Queensland 2002–2005^*^	Serology	- Barrett’s Oesophagus - Controls	18–79	296 390	9.5 18.7
Fabis Pedrini et al., 2015 Western Australia 2007–2011	Serology	- Multiple Sclerosis - Community controls	23–69 (Mean 43.7)	299 299	15.1 21.4
Van der Poorten et al., 2018 New South Wales 2016–2018	Rapid Urease	- Common variable immunodeficiency (CVID) - Controls	18–82 (mean 51) 18-74 (mean 48)	50 40	8.0 (*n* = 4) 8.1 (*n* = 3)
Edwards et al., 1991 New South Wales not stated	Histology Serology	- Male AIDS patients with GI dysfunction - Controls - HIV-negative dyspeptic patients	18–59	201 702 137	3.0 21.7 59.1
Mitchell et al., 2008 Victoria not stated	Serology (ELISA & Immunoblot)	- Cardia cancer - Controls for cardia cancer patients - Non-cardia cancer - Controls for non-cardia cancer patients	42–69	18 69 34 134	33/44 35/39 79/94 63/63 (ELISA/Immunoblot)
Hunt et al., 2014 (Conference abstract) New South Wales not stated	Histology	Coeliac disease	not stated	53	5.6
**STUDY DESIGN: CROSS-SECTIONAL**					
Hardikar et al., 1991 Victoria May 1986–January 1989	Culture Histology Rapid Urease Serology	Endoscopy patients	1 month–26 years	363	7.7
Mitchell et al., 1993b New South Wales 1987–1991	Histology Rapid Urease Serology	- Children - Adults	6mths–18yrs 16–78	227 258	14.1 53.9
Chow et al., 1995 Victoria 1990	Serology	Adults of Chinese descent	25+	328	59.5
Hardikar & Grimwood, 1995 Victoria 1991	Serology	Children attending for minor elective surgery	0–14	147	14.3
Lin et al., 1998a Victoria 1991	Serology	Subjects with Anglo-Celtic names	20–80	273	38.0
Mollison et al., 1994 Central Australia 1991–1992	Histology (Giemsa stain)	Aboriginal endoscopy patients	19–80 (mean 43)	64	75.0
Peach, Bath & Farish, 1998 Victoria 1992	Serology	Ballarat health survey participants	adults	312	30.1
Peach, Pearce & Farish, 1997 Victoria 1994 - 1995	Serology	Ballarat health survey participants	adults	217	30.6 (age standardised prevalence)
Xia et al., 2001 New South Wales 1994 & 1998	Histology Rapid Urease	Endoscopy patients - 1994 - 1998	51.1 ± 19.0 51.4 ± 17.1	202 298	39.1 29.9
Lee, Windsor & Marshall, 2001 (Conference abstract) Western Australia 1994	Serology Urea Breath Test Molecular typing	- General married population - Spouse Hp+	not stated	1000 200	25.1 41.5
Xia et al., 2000b New South Wales 1996–1998	Serology Culture Rapid Urease Histology	Dyspepsia or reflux symptoms	18–86 (mean 52.0)	277	41.5
Henry & Batey, 1998 New South Wales Jan–Oct 1997	Rapid Urease Serology Urea Breath Test	Duodenal ulcer	Mean 58	125	55
Ho et al., 2001 Western Australia 1997–1999	Rapid Urease Culture Histology PCR	Sudden Infant Death Syndrome	4–52 weeks	9	0
Samarasam, Roberts-Thomson & Brockwell, 2009 Tasmania 1997–2007	Histology Rapid Urease	Fundic Gland Polyps	21 - 89	120	3.0
Mollison et al., 2000 Western Australia 1998–1999	Culture and gram stain	Endoscopy patients	18+	434	29.5
McDonald et al., 2004 Northern Territory 1999–2000	Serology	Adult Aboriginal community	18+	212	72.0
Wallace, Webb & Schluter, 2002 Queensland 1999–2000	Serology Faecal antigen	Adults with intellectual disability, institutionalised: - Long term - Previously - Never	17+ 35.8 ± 8.9 39.1 ± 12.2 29.4 ± 8.7	76 53 39	86.8 79.2 43.6
Endall et al., 2020 Tasmania 1982–2018	Serology	Patients with Multiple Endocrine Neoplasia Type 1 (MEN1)	Median 44	95	35.8
Moujaber et al., 2008 National 2002	Serology	Laboratory sample	1–59	2413	15.1
Ritchie et al., 2009 Northern Territory 2002–2004	Urea Breath Test Faecal antigen	Aboriginal children with acute diarrhoeal disease.	4 months– 2 years	52	44.2
Pandeya, Whiteman & Australian Canc Study, 2011 National 2002–2005	Serology	Controls matched to oesophageal cancer cases	18–79	1355	22.3 (Standardised by age and sex to the Australian population: 15.5)
Windsor et al., 2005 Western Australia 2003–2004	Urea Breath Test	Aboriginal patients: - Urban - Remote	3–75 2–90	250 270	60 91
Lam, Trinh & Wilson, 2006 New South Wales 2003–2004	Histology Rapid Urease	Symptomatic gastroscopy patients	13–89	179	31.3
Gibney et al., 2009 Victoria 2003–2006	Gastroscopy Urea Breath Test Serology	Immigrants from sub-Saharan Africa and Sudan	16–76	58	60.3
Chaves et al., 2009 Victoria 2004–2008	Faecal antigen Urea Breath Test Serology	Burmese refugees	16–86	41	80.5
Cherian et al., 2008 Western Australia 2006	Faecal antigen - Monoclonal (MFAT) - Immuno- chromatographic technique (ICT) Serology	African refugees	<16 (mean 7.9)	MFAT: 182 ICT: 176 Serology: 192	81.9 67.0 47.4
Mutch et al., 2012 Western Australia 2006–2008	Serology	Refugee children	2 months– 17 years	1026	20.1
Kane, Shenstone & Katelaris, 2009 (Poster abstract) New South Wales 2008^*^	Serology	Patients on Non-Steroidal Anti-inflammatory Drugs (NSAIDs)	>60	50	40
Hiew et al., 2012 New South Wales August 2008-April 2009	Serology	Percutaneous coronary intervention patients	64.4 ± 11	245	37
Johnston, Smith & Roydhouse, 2012 Northern Territory 2009–2010	Serology Urea Breath Test Faecal antigen	Symptomatic refugee patients	not stated for this group	18	50.0
Abdul Rahim et al., 2017 South Australia 2010–2013	Faecal antigen	Newly arrived migrants	0–82	922	21.5
Benson, Rahim & Agrawal, 2017 South Australia 2010–2013	Faecal antigen	Newly arrived refugee children	0–19	460	21.0
Wise, Lamichhane & Webberley, 2019 Western Australia 2010–2015	Urea Breath Test	All patients with UBT test results	1–98	77552	22.0
Buckle et al., 2018 (Conference abstract) Victoria 2012–onwards	Histopathology	Gastric biopsy specimens	Not stated	Total: 959 Patients born in Asia: 102	10.1 18.0
Vaughan & Metz, 2017 (Conference abstract) Victoria Jan 1, 2015–Dec 31, 2016	Not stated	Patients with a new gastric ulcer diagnosis	Not stated	101	26.7
Sharma & Dowling, 2018 (Conference abstract) Victoria Oct 2017–April 2018	Histopathology	Having routine diagnostic gastroscopy	>50 median = 66	80	6.0
Mitchell et al., 1988 New South Wales Not stated	Serology	-Upper GI symptoms - Controls	Not stated 0–62	189 785	Histology: 63.5 Serology: 65.6 20.0
Dwyer et al., 1988b Victoria & Northern Territory not stated	Serology	- Aboriginal participants - ’Healthy’ white participants - Duodenal ulcer patients	10–59	274 144 142	0.7 14.6 62.7
Dwyer et al., 1988a Victoria not stated	Serology	Refugees - Vietnamese - El Salvadorean - Ethiopian	10–60+	190 75 74	18.4 40.0 43.2
Mitchell, Lee & Carrick, 1989 New South Wales not stated	Serology	Gastroenterologists Gastroenterology nurses General practitioners Blood donors (controls)	28–65 25–60 32–65 25–65	33 68 35 715	51.5 19.1 28.6 21.5
Lin et al., 1991 (Conference abstract) Victoria not stated	Serology	- Chinese - Japanese - Caucasian	24–84 (mean 45) 29–50 (mean 39) 20–77 (mean 52)	341 85 98	59.5 60.0 30.6
Clancy et al., 1994 New South Wales not stated	Histology Rapid Urease Serology Salivary antigen	Dyspeptic endoscopy patients	22–83 (mean 58.9)	134	28.4
Lin et al., 1994 Victoria not stated	Serology	- Gastroenterologists - Controls for Gastroenterologists - General internists - Controls for General internists - Gastroenterology nurses - Controls for Gastroenterology nurses - General nurses - Controls for general nurses	31–73 28–67 23–60 22–50	39 195 25 40 107 115 42 120	69.2 36.9 40.0 37.5 16.8 27.8 19.0 24.2
Borody, Andrews & Shortis, 1996 New South Wales not stated	Rapid Urease Histology	Dyspepsia	52.7 ± 15.7	203	35.0
Leong et al., 1998 (Conference abstract) Victoria not stated	Serology	-Anaesthetists & Anaesthetist trainees - Representative normal population (no details given)	26–79	84 239	27.4 36.8
Lin et al., 1998b Victoria not stated	Serology	- Dentists - Controls for Dentists - 1st year Dental students - 5th year Dental students - Controls for Dental students - Dental nurses - Controls for Dental nurses	42 ± 11.2 19 ± 1.2 24 ± 1.5 32 ± 9.4	92 187 30 33 14 40 108	22.8 33.2 16.7 18.2 17.6 17.6 30.6
Talley et al., 1998 New South Wales & Victoria not stated	Urea Breath Test	Patients with dyspepsia: - Melbourne - Sydney	25–85	65 45	58.5 46.7
Peach, Bath & Farish, 1999 Victoria not stated	Serology	Ballarat health survey participants	adults	324	30.2
Robertson, Cade & Clancy, 1999 Victoria not stated	Serology (Rapid Whole Blood Test)	- Intensive care patients - Controls for intensive care patients - Intensive care nurses - Controls for nurses	19–88 23–45	100 500 100 246	67 39 40 19
Xia et al., 2000a New South Wales not stated	Serology Culture Rapid Urease Histology	Dyspepsia and reflux symptoms	17–85	209	40.2
Peach & Barnett, 2001 Victoria not stated	Serology	Ballarat health survey participants	19–87	248	35.5
Kaffes et al., 2003 New South Wales not stated	Serology	Well, older adults (aged ≥65)	≥65 (mean 75)	220	42.3
Robertson et al., 2003 Victoria not stated	Serology (Rapid Whole Blood Test)	Consecutive blood donors	16–71	500	31.4
Ren et al., 2005 New South Wales not stated	Histology Rapid Urease Serology	Dyspepsia	16–87	168	32.1
Bergmann-Hug et al., 2010 (Poster abstract) South Australia not stated	Serology	Chronic idiopathic urticaria	17–73	27	22.2

**Notes.**

* Data collection date determined by contacting the author.

Studies were performed in all Australian states and in the Northern Territory, with more than 70% of the publications reporting findings from Victorian, New South Wales or Western Australian populations. The number of participants ranged from nine (Ho et al., 2001) to over 70,000 (Wise, Lamichhane & Webberley, 2019). Around 50% of the studies included 100–500 participants. Over a third of the studies investigated patients with gastrointestinal (GI) symptoms or conditions. Patients with non-GI related conditions, for example coronary heart disease (Coles et al., 2003), diabetes (Schimke et al., 2009), sudden infant death syndrome (SIDS) (Ho et al., 2001), multiple sclerosis (Pedrini et al., 2015) and HIV/AIDS (Edwards et al., 1991) were also commonly investigated. Specific cultural groups studied included both urban and rural Aboriginal populations (8.0% of the included publications) (Windsor et al., 2005; Dwyer et al., 1988b; Pringle et al., 2015; Mollison et al., 1994; McDonald et al., 2004; Ritchie et al., 2009), newly arrived migrants (10.6% of included studies) (Dwyer et al., 1988a; Gibney et al., 2009; Chaves et al., 2009; Cherian et al., 2008; Mutch et al., 2012; Johnston, Smith & Roydhouse, 2012; Abdul Rahim et al., 2017; Benson, Rahim & Agrawal, 2017), ethnic groups such as members of the Chinese population of Melbourne (2.6% of included studies) (Chow et al., 1995; Lin et al., 1991), and institutionalised populations (2.6% of included studies) (Lambert et al., 1995; Wallace, Webb & Schluter, 2002). Several studies investigated groups of health professionals, hypothesised to be at greater risk of contracting *H. pylori*, including dentists (Lin et al., 1998b) gastroenterologists (Lin et al., 1994) and nurses (Robertson, Cade & Clancy, 1999). Fourteen of the included papers (19%) estimated *H. pylori* prevalence in children (Windsor et al., 2005; Moujaber et al., 2008; Dwyer et al., 1988a; Dwyer et al., 1988b; Mitchell et al., 1993a; Hardikar et al., 1996; Hardikar et al., 1991; Mitchell et al., 1993b; Hardikar & Grimwood, 1995; Ho et al., 2001; Ritchie et al., 2009; Cherian et al., 2008; Mutch et al., 2012; Benson, Rahim & Agrawal, 2017).

A range of different diagnostic methods were used to determine *H. pylori* presence in the included studies. Histology, rapid urease and culture are invasive tests performed on tissue samples collected by endoscopy. Non-invasive tests include serology, urea breath test (UBT) and faecal antigen (FA) test. Among the studies included in this review, serology was by far the most common method used to detect presence of *H. pylori* infection, used in 56 (75%) studies. Serology and histology have been used throughout the study period. The earliest study using UBT as the diagnostic method was published in 1994 (Borody et al., 1994) and FA was first used for *H. pylori* testing in an Australian epidemiological study in 2002 (Wallace, Webb & Schluter, 2002).

The estimated prevalence of *H. pylori* in included studies was wide-ranging, among diverse populations, from 0% in SIDS babies in 1997–1999 (Ho et al., 2001) to 91% in Aboriginal community members in 2003–2004 (Windsor et al., 2005). Estimated prevalence among children ranged from 0% in SIDS babies (Ho et al., 2001) to 85% in a group of Aboriginal children (Windsor et al., 2005). In 2002, Moujaber and colleagues estimated that the *H. pylori* prevalence was 7.8% among children in the general population aged 1 to 19 years (Moujaber et al., 2008). Prevalence was similarly low among patients with conditions including oesophageal cancer (Whiteman et al., 2010), Barrett’s oesophagus (Thrift et al., 2012) and fundic gland polyps (Samarasam, Roberts-Thomson & Brockwell, 2009). Male AIDS patients (Edwards et al., 1991) and females with multiple sclerosis (Pedrini et al., 2015) were also found to have a low prevalence of *H. pylori* infection. Gastric cancer patients (Dugué et al., 2019; Mitchell et al., 2008), institutionalised individuals (Lambert et al., 1995; Wallace, Webb & Schluter, 2002), refugees (Chaves et al., 2009; Cherian et al., 2008; Johnston, Smith & Roydhouse, 2012) and Aboriginal and Torres Strait Islander populations (Windsor et al., 2005; Mollison et al., 1994; McDonald et al., 2004) typically had high prevalence of *H. pylori* infection. Recent prevalence estimates are lacking for vulnerable groups. The most recent prevalence estimates available for these groups are: 60% in an urban Aboriginal population in 2003–2004; 91% in a non-urban Aboriginal population at the same time; 21.5% in a refugee population in metropolitan South Australia in 2010–2013; 86.8% in long-term institutionalised and 79.2% in previously institutionalised adults with intellectual disability in 1999–2000; 31.6% in adults aged over 70 in 2002–2005; and 69.2% in gastroenterologists studied in 1994.

**Figure 2:**
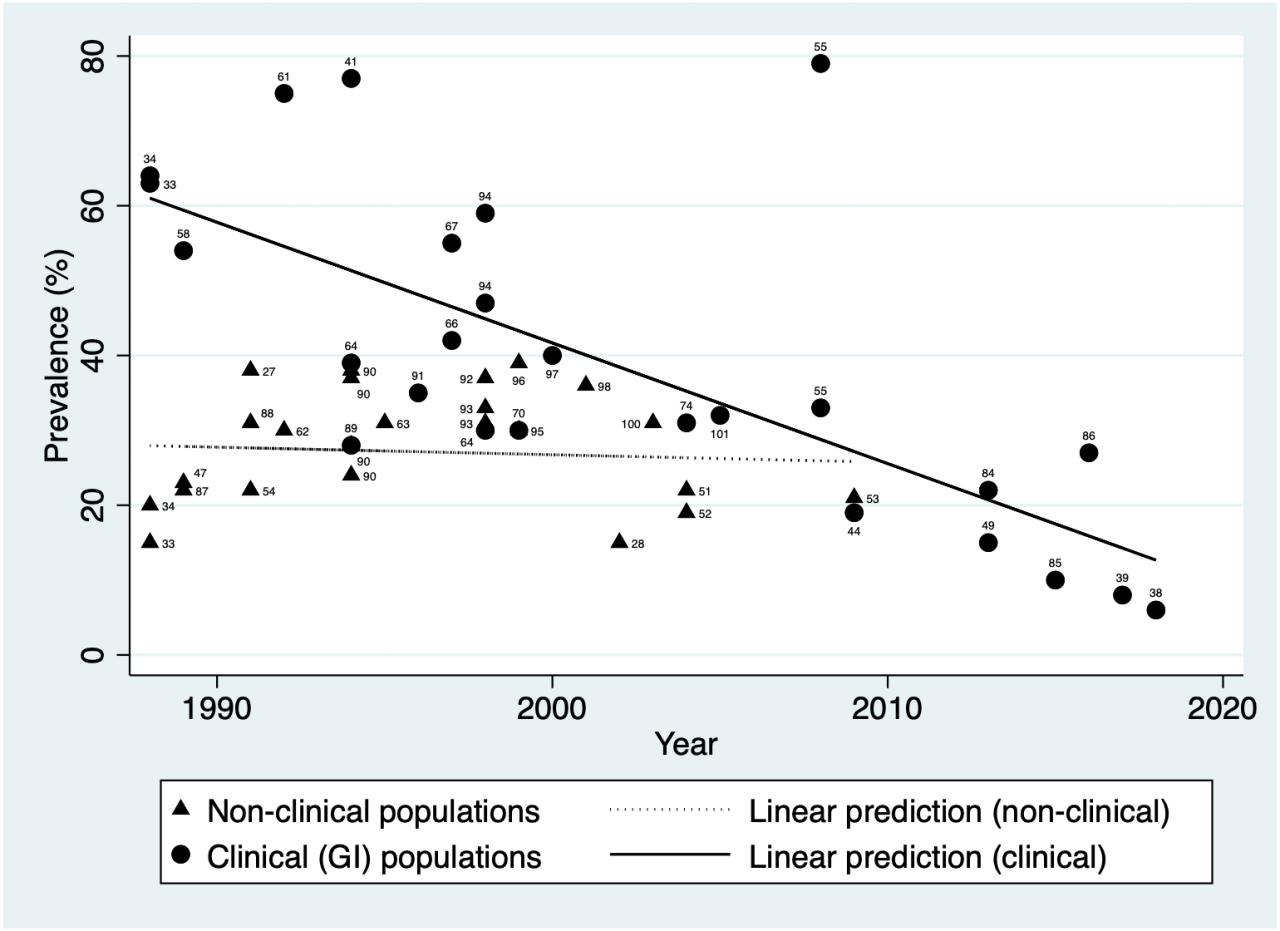
*H. pylori* prevalence over time in populations with gastrointestinal conditions and in non-clinical populations in Australia. Data labels indicate source reference.

*H. pylori* prevalence estimated in general population studies ranged from 38.0% in 1991 (Lin et al., 1998a) to 15.1% in 2002 (Moujaber et al., 2008). In addition to population-based studies, a number of studies included control groups such as blood donors. Figure 2 illustrates prevalence over time in non-clinical populations (excluding studies that looked only at children) and indicates a stable prevalence between 1988 and 2009 (Coefficient = −0.10, 95% CI [−0.66–−0.46]). This is shown alongside the pronounced downward trend seen in clinical populations with gastrointestinal conditions or symptoms (Coefficient = −1.61, 95% CI [−2.26–−0.97]).

Prevalence estimate ranges by birth decade were determined from the studies that reported prevalence estimates in general populations by age-group (Pandeya, Whiteman & Australian Canc Study, 2011; Lin et al., 1998a; Moujaber et al., 2008; Dwyer et al., 1988b; Mitchell et al., 1988), and are presented in Table 2. Observation of these prevalence ranges across birth decades appears to indicate lower prevalence with successive birth cohorts.

**Table 2:** *H. pylori* Australian prevalence estimate ranges by decade of birth.

**Decade of birth**	**Prevalence range (%)**
Earlier than 1920	53.0
1920s	20.0–46.0
1930s	26.0–37.0
1940s	16.7–27.0
1950s	11.7–24.0
1960s	18.0–18.4
1970s	5.0–12.4
1980s	4.0–10.0
1990s	4.0–8.3

## Discussion

The aim of this review was to describe the scope of studies to have documented the prevalence of *H. pylori* in Australia with regard to study characteristics such as study design, geographic region, population characteristics, and diagnostic methods, and to summarise the estimated prevalence in the included studies according to person characteristics and time. This review has compiled the most comprehensive collection of Australian-based *H. pylori* prevalence data to date.

*H. pylori* prevalence estimated in Australian general population studies ranged from 38.0% in 1991 (Lin et al., 1998a) to 15.1% in 2002 (Moujaber et al., 2008), but prevalence has varied dramatically across population sub-groups. In Australia, it appears from the data summarised from included studies in this review that there may have been a decrease in prevalence in recent decades, which may be more marked in populations with GI symptoms and conditions than in the general population. Whether this is a true difference is unknown, as this study did not consider the quality of included studies. However, several international studies have similarly claimed a recent decrease in *H. pylori* prevalence in clinical populations. Leow et al. (2016) reported that *H. pylori* prevalence decreased in first-time gastroscopy patients in a single medical centre in Malaysia, from 51.7% in 1989–1990 to 11.1% in 2009–2010. Kamada et al. (2015) collected data from gastric biopsies performed in Honshu, Japan and reported that *H. pylori* prevalence decreased from 74.7% in the 1970s to 35.1% in the 2010s. However, no studies have compared clinical and non-clinical populations in the same country. Our observation of a potentially smaller decrease in prevalence in non-clinical populations is novel. The apparent larger decrease in prevalence among clinical populations may be attributed to greater levels of diagnostic testing and treatment for *H. pylori* infection among those with gastrointestinal symptoms compared to infected individuals who are asymptomatic. Aro et al. (2006) and Bae et al. (2018) reported that peptic ulcer and gastric cancer were present even in asymptomatic populations so a stable prevalence in non-clinical populations may be of concern.

Analysis of the included studies found that while no longitudinal or comparable studies have been performed, some evidence for a decrease in prevalence comes from examining the data from the point of view of prevalence by birth year using the studies that have estimated prevalence by age-group. This indicates a clear cohort effect. Evidence from this review suggests that *H. pylori* prevalence in Australia was much lower in the early 21st century than in the first half of the 20th century. This observation may be explained by a decrease in childhood acquisition rates in line with a improvements to living conditions, such as household size (a measure of ‘crowding’), over the 20th century. Information from the Australian Institute of Health and Welfare shows that average household size decreased from 4.5 persons in 1911 to 2.6 persons in 2016 (Australian Institute of Family Studies, 2021). Interestingly, a plateau in *H. pylori* prevalence has been observed in the early 21st century among children in Holland (den Hoed et al., 2011). While it appears there may have been a similar plateau in childhood acquisition of *H. pylori* infection in Australia, potentially driving the decrease in prevalence from the first half of the 20th century to the early 21st century, this decrease may not continue into the future if a decrease in adult-acquired infections does not also follow and if further improvements to living conditions are not made. For instance, while data from the Australian Institute of Health and Welfare shows average household size decreased from 4.5 to 2.6 persons between 1911to 2016, there was no change from 2001 to 2016 (Australian Institute of Family Studies, 2021). Further, evidence from studies of institutionalised adults (Lambert et al., 1995; Wallace, Webb & Schluter, 2002), gastroenterologists (Mitchell, Lee & Carrick, 1989; Lin et al., 1994) and married couples (Lee, Windsor & Marshall, 2001), indicate that acquisition in adulthood is possible, and there are not sufficient data to determine whether the proportion of adult-acquired infections is decreasing over time and/or whether the proportion of adult-acquired infections has an impact on changes in population prevalence either historically or into the future. However, the potential that overall *H. pylori* prevalence is stabilising in Australia remains a possibility that is not currently being discussed in the (Australian) literature, with associated issues of anti-microbial resistance in eradication treatment, and risks of peptic ulcers and gastric cancer.

Whether or not the overall prevalence of *H. pylori* has decreased in Australia, it is important to note that high prevalence has been reported in marginalised and vulnerable population sub-groups in Australia such as Indigenous (Windsor et al., 2005; Pringle et al., 2015; McDonald et al., 2004), migrant (Chow et al., 1995; Lin et al., 1991), refugee (Gibney et al., 2009; Chaves et al., 2009; Cherian et al., 2008; Mutch et al., 2012; Abdul Rahim et al., 2017; Benson, Rahim & Agrawal, 2017) and institutionalised populations (Lambert et al., 1995; Wallace, Webb & Schluter, 2002), the elderly (Pandeya, Whiteman & Australian Canc Study, 2011; Lin et al., 1998a; Kaffes et al., 2003) and health professionals with higher exposure to *H. pylori* positive patients (Mitchell, Lee & Carrick, 1989; Lin et al., 1994), consistent with worldwide studies (Jones et al., 2012; Eusebi, Zagari & Bazzoli, 2014; Fagan-Garcia et al., 2019; Pabla et al., 2020; Kheyre et al., 2018). This review indicates that recent data for these groups in Australia are lacking. As the number and proportion of older Australians increases (Australian Institute of Health and Welfare, 2021b), it is important to know whether prevalence remains high in this population group. Data from the Australian Institute of Health and Welfare indicate that gastric cancer incidence declined from 9.3−7.5 cases per 100,000 persons between 1998 and 2013 among non-Indigenous Australians, as did gastric cancer mortality (6.1−3.9 deaths per 100,000 persons, 1998–2015). However, rates in Australian Indigenous populations have remained stable over time (gastric cancer incidence: 10.0–14.3 cases per 100,000 persons, 1998–2013; gastric cancer mortality: 6.7−8.8 deaths per 100,000 persons, 1998–2015) (Australian Institute of Health and Welfare, 2021a). High prevalence of *H. pylori* infection and gastric cancer are also seen in other Indigenous populations, for example in New Zealand (Signal et al., 2020) and Canada (Jones et al., 2012). Management of *H. pylori* infection and associated disease in these at-risk groups requires up to date and accurate information. A 2005 report of very high (91%) prevalence within an Aboriginal community (Windsor et al., 2005) sparked a call for more research by others (Talley, 2005). As far as we can tell this is yet to eventuate.

Prevalence estimation in the general Australian population is challenging due to the populations recruited to studies included in this review. For instance, blood donors, who are commonly recruited for epidemiological studies, have been shown to poorly represent *H. pylori* prevalence in the general population, particularly in relation to older age groups. A study from Sweden (Sörberg, Nyrén & Granström, 2003), for example, showed that older participants who were *H. pylori* positive were less likely to be regular blood donors, possibly because blood taking was more likely to make them feel unwell, compared to *H. pylori* negative participants. Also related to age, the sero-surveillance survey included in this review (Moujaber et al., 2008) only included participants aged up to 59 years, so is likely to have underestimated population prevalence. There is also no information available about the likely socio-economic profile of the sera used in that study. Some research shows that non-participants in control groups are more likely to be of lower socio-economic status than participants (Pandeya et al., 2009). Since *H. pylori* positivity is inversely associated with socio-economic status, this may also affect prevalence estimation.

With regard to diagnostic testing, serology was the most commonly used test in the studies included in this review, consistent with world-wide epidemiological studies (Zamani et al., 2018). It has been noted that serological tests are commonly used for epidemiological studies (Katelaris et al., 2021), as they are widely available and inexpensive (Tshibangu-Kabamba et al., 2021). However, antibodies to *H. pylori* can remain at high levels for some time after eradication of the infection (Ricci, Holton & Vaira, 2007); thus, using serological data may lead to misclassification of *H. pylori* presence and absence, leading to a lack of confidence in estimates of prevalence in serological studies. This is reflected in Australian clinical guidelines for diagnosis of *H. pylori*, which recommend the use of UBT or FA tests over serology (Mitchell & Katelaris, 2016; Stenström, Mendis & Marshall, 2008).

### Strengths and limitations

This study has followed the rigorous and globally accepted methodologies for scoping reviews. Therefore, we can be confident that every possible effort was made to include all relevant research. The main limitation is that no quality appraisal of the included studies was undertaken. Although this is not strictly necessary for scoping reviews, it does mean that some included studies may be of lower standard.

## Conclusion

This scoping review has provided, to our knowledge, the first structured review of studies reporting prevalence of *H. pylori* in Australia. A wide range of studies was reviewed based on data collected over a 50-year period (1969 to 2018), including diverse study designs, population groups, geographic regions within Australia, and diagnostic methods. The summary of *H. pylori* prevalence estimates over time in this review points to a decrease in *H. pylori* prevalence in Australia, particularly among clinical populations; however, it appears that prevalence in the general population without gastrointestinal symptoms or disease has remained relatively stable over time. While this novel study adds to current knowledge, there are several specific population groups for whom further research is warranted. For instance, it is unknown whether there is enduring inequity in patterns of prevalence across vulnerable sub-groups of the Australian population, specifically, older Australians and Aboriginal populations. Given the stable rates of gastric cancer among Australian Aboriginal populations, a decrease in *H. pylori* prevalence over time is unlikely to have occurred; however, without the data to evidence this, interventions to improve infection rates, and morbidity and mortality from resultant illnesses such as gastric cancer, may be limited. A new national survey using UBT or FA would also be a useful addition to our understanding of the prevalence and epidemiology of *H. pylori* in Australia, given the limitations in accuracy of serology tests.

##  Supplemental Information

10.7717/peerj.13430/supp-1 Supplemental Information 1 PRISMA Checklist

10.7717/peerj.13430/supp-2 Supplemental Information 2 Medline search strategy

## Abbreviations

ABS: Australian Bureau of Statistics
FA: Faecal antigen
MALT: Mucosa-associated lymphoid tissue
PRISMA: Preferred Reporting Items for Systematic Reviews and Meta-Analyses
UBT: Urea Breath Test

## Additional Information and Declarations

### Data Availability

The following information was supplied regarding data availability: The data collected for this review is available in Table 1.
