# Supplementary material for: Epidemiology of Helicobacter pylori in Australia: a scoping review
Source: PeerJ. 2022 May 31;10:e13430. doi: 10.7717/peerj.13430 (PMC9165601; doi:10.7717/peerj.13430)
Supplement: Supplemental Information 2 [file peerj-10-13430-s002.docx]

**Additional File 1**

**Medline search strategy**

1. *Helicobacter pylori*/

2. (*Helicobacter pylori** or *H? pylori** or *Campylobacter pylori**).mp. [mp=title, abstract, original title, name of substance word, subject heading word, keyword heading word, protocol supplementary concept word, rare disease supplementary concept word, unique identifier, synonyms]

3. 1 or 2

4. exp Australia/

5. (australia* or tasmania* or victoria* or new south wales or queensland* or northern territor* or christmas island* or canton island* or enderbury island* or melbourn* or sydney or adelaid* or perth or hobart or canberra or brisbane or darwin).mp. [mp=title, abstract, original title, name of substance word, subject heading word, keyword heading word, protocol supplementary concept word, rare disease supplementary concept word, unique identifier, synonyms]

6. 4 or 5

7. prevalence/

8. (prevalen* or infection rate* or proportion* or frequenc* or occurrence* or likelihood* or probabilit*).mp. [mp=title, abstract, original title, name of substance word, subject heading word, keyword heading word, protocol supplementary concept word, rare disease supplementary concept word, unique identifier, synonyms]

9. Epidemiology/

10. epidemiolog*.mp.

11. risk factors/

12. ("population? at risk" or risk factor?).mp. [mp=title, abstract, original title, name of substance word, subject heading word, keyword heading word, protocol supplementary concept word, rare disease supplementary concept word, unique identifier, synonyms]

13. exp Cohort Studies/

14. (follow up stud* or follow?up stud* or longitudinal stud* or longitudinal survey* or prospective stud* or retrospective stud* or cohort stud* or cohort analys?s or con?current stud* or incidence stud* or cross?section* stud*).mp. [mp=title, abstract, original title, name of substance word, subject heading word, keyword heading word, protocol supplementary concept word, rare disease supplementary concept word, unique identifier, synonyms]

15. population surveillance/

16. (Population Surveillance or Sentinel Surveillance or Public Health Surveillance or general population* or screen*).mp. [mp=title, abstract, original title, name of substance word, subject heading word, keyword heading word, protocol supplementary concept word, rare disease supplementary concept word, unique identifier, synonyms]

17. Asymptomatic Infections/

18. (a?symptomatic infection* or sub?clinical infection*).mp. [mp=title, abstract, original title, name of substance word, subject heading word, keyword heading word, protocol supplementary concept word, rare disease supplementary concept word, unique identifier, synonyms]

19. 7 or 8 or 9 or 10 or 11 or 12 or 13 or 14 or 15 or 16 or 17 or 18

20. 3 and 6 and 19

21. (sydney system* or sydney scor* or sydney classification* or sydney scor* system* or sydney protocol* or sydney criteri* or sydney scale*).mp. [mp=title, abstract, original title, name of substance word, subject heading word, keyword heading word, protocol supplementary concept word, rare disease supplementary concept word, unique identifier, synonyms]

22. (sydney strain or strain sydney).mp. [mp=title, abstract, original title, name of substance word, subject heading word, keyword heading word, protocol supplementary concept word, rare disease supplementary concept word, unique identifier, synonyms]

23. 21 or 22

24. 20 not 23
